# What Do Patients Say About Doctors Online? A Systematic Review of Studies on Patient Online Reviews

**DOI:** 10.2196/12521

**Published:** 2019-04-08

**Authors:** Y Alicia Hong, Chen Liang, Tiffany A Radcliff, Lisa T Wigfall, Richard L Street

**Affiliations:** 1 Department of Health Administration and Policy George Mason University Fairfax, VA United States; 2 School of Public Health Texas A&M University College Station, TX United States; 3 Arnold School of Public Health University of South Carolina Columbia, SC United States; 4 Department of Health Kinesiology Texas A&M University College Station, TX United States; 5 Department of Communication Texas A&M University College Station, TX United States

**Keywords:** patient review websites, patient online review, systematic review

## Abstract

**Background:**

The number of patient online reviews (PORs) has grown significantly, and PORs have played an increasingly important role in patients’ choice of health care providers.

**Objective:**

The objective of our study was to systematically review studies on PORs, summarize the major findings and study characteristics, identify literature gaps, and make recommendations for future research.

**Methods:**

A major database search was completed in January 2019. Studies were included if they (1) focused on PORs of physicians and hospitals, (2) reported qualitative or quantitative results from analysis of PORs, and (3) peer-reviewed empirical studies. Study characteristics and major findings were synthesized using predesigned tables.

**Results:**

A total of 63 studies (69 articles) that met the above criteria were included in the review. Most studies (n=48) were conducted in the United States, including Puerto Rico, and the remaining were from Europe, Australia, and China. Earlier studies (published before 2010) used content analysis with small sample sizes; more recent studies retrieved and analyzed larger datasets using machine learning technologies. The number of PORs ranged from fewer than 200 to over 700,000. About 90% of the studies were focused on clinicians, typically specialists such as surgeons; 27% covered health care organizations, typically hospitals; and some studied both. A majority of PORs were positive and patients’ comments on their providers were favorable. Although most studies were descriptive, some compared PORs with traditional surveys of patient experience and found a high degree of correlation and some compared PORs with clinical outcomes but found a low level of correlation.

**Conclusions:**

PORs contain valuable information that can generate insights into quality of care and patient-provider relationship, but it has not been systematically used for studies of health care quality. With the advancement of machine learning and data analysis tools, we anticipate more research on PORs based on testable hypotheses and rigorous analytic methods.

**Trial Registration:**

International Prospective Register of Systematic Reviews (PROSPERO) CRD42018085057; https://www.crd.york.ac.uk/PROSPERO/display_record.php?RecordID=85057 (Archived by WebCite at http://www.webcitation.org/76ddvTZ1C)

## Introduction

People have increasingly turned to the internet to share their clinical experience and make comparisons of physicians and medical treatments [1,2]. Hundreds if not thousands of patient online reviews (PORs) appear daily on the crowdsource platforms of patient review websites (PRWs) and carry growing influence in patients’ medical decision making [1-4]. In the earlier debates of PORs, some physicians expressed skepticism; they worried that most PORs were posted by begrudged patients who were not able to assess the technical quality of health care delivery [5]. Furthermore, physicians are unable to refute a negative review without jeopardizing patient confidentiality [6]; and it is nearly impossible to verify if the comments were left by actual patients [3]. Also, even with an increasing number of PORs, most rated physicians average a handful of ratings, which is unlikely to reflect the full range of impressions made by a physician who sees hundreds of patients each year [6]. Proponents of PORs, however, argue that patients are like consumers of other services and therefore have a right to express their opinions about services they pay for, and PORs provide timely and direct customer feedback [3,6,7].

Despite the ongoing debates on whether PORs can improve the quality of care [8,9], the number of PORs has grown exponentially in the past decade [1,10,11]. A recent national survey in the United States revealed that 59% of participants reported PORs were very important or somewhat important when choosing a physician, though PORs were endorsed less frequently than other factors such as word of mouth from family and friends and whether the physician accepted one’s insurance [2].

The proliferation of PORs and popularity of PRWs has happened in 2 somewhat overlapping contexts. Of these, 1 is that the ubiquitous internet access has facilitated online consumer behaviors, featured by “electronic word of mouth” [12]. People go online to rate any product or service they purchase and check online ratings before making any purchase. Health care consumer behaviors, though lagging other consumer behaviors, are rapidly catching up [3]. The other context is the movement of patient empowerment and self-determination of medical care, alongside the more recognized importance of patient experience and patient satisfaction in evaluating health care quality [13,14]. For example, the Center for Medicare and Medicaid Services (CMS) has a set of Core Quality Measures for Healthcare, and “patient experience” is one of the 7 critical domains [15]. Traditional government- or health care organization (HCO)–initiated surveys have incorporated patient-reported outcome measures in their routine questionnaires of quality measures, but it takes years to conduct surveys and analyze the data, and few patients have access to or understand these data [13]. Within such contexts, PORs have become a consumer-driven alternative that can provide almost instant feedback of health care experience.

The increasing weight of PORs in patients’ health care decision making has led to a growing number of research studies on PORs and PRWs [1,11]. Some scholars have advocated for giving more scientific values to PORs [7,16]. Others have evaluated the quality of PRWs and examined public perceptions and use of PRWs [2,10,17,18]. They concluded that the research on and usage of PRWs was limited [17,18]. To date, no systematic review of POR studies was available. Accordingly, we conducted a systematic review with the aims to synthesize existing studies on PORs by summarizing study characteristics, research design, analytical methods, and major findings. We have depicted the trend of POR research, identified literature gaps, and made recommendations for future research.

## Methods

### Inclusion and Exclusion Criteria

On the basis of the research objectives mentioned above, we listed the following search criteria before we started the literature search. The inclusion criteria were as follows: (1) studies that focused on PORs of physicians or hospitals, (2) studies that reported qualitative or quantitative results from analyses of PORs, and (3) peer-reviewed studies written in English. The exclusion criteria were (1) studies that did not report empirical outcomes from analyses of PORs and (2) editorials, reviews, or commentaries. Excluded studies were, for example, focused on physicians’ responses to online reviews [19], reported innovative methods for analyzing PORs without reporting the analytical results [20,21], or focused on characteristics of the patients who had used PRWs without reporting POR-related outcomes [2,22].

### Data Sources and Selection

**Figure 1:**
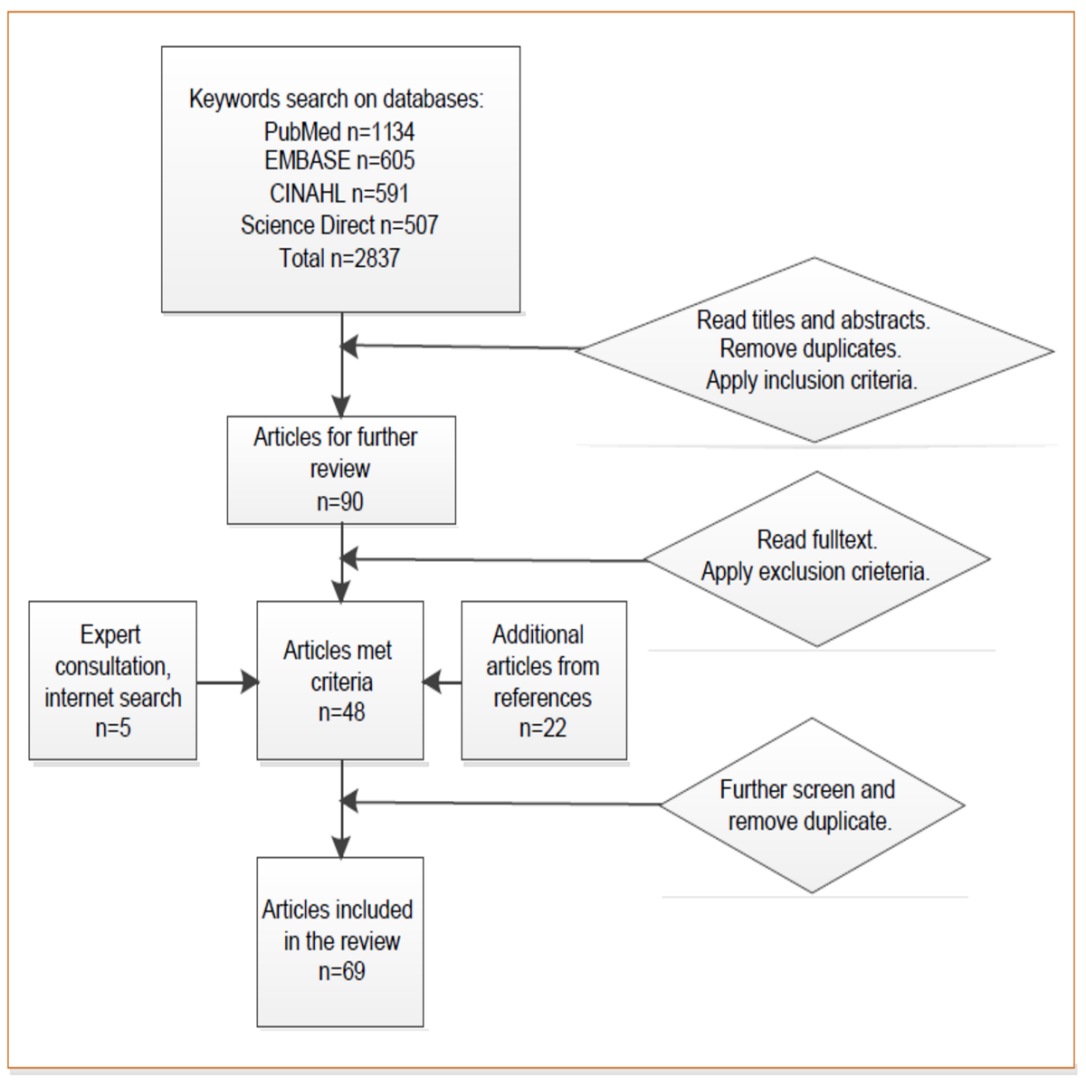
Flow chart of the literature search and article retrieval.

Following the principles of Preferred Reporting Items for Systematic Reviews and Meta-Analyses [23], we searched the major databases of PubMed, EMBASE, CINAHL, and Science Direct in January 2019. Search terms from previously published studies were used [17,18,24], including rating sites (websites), review sites (websites), online reviews (ratings), doctor (physician and hospital) ratings, and patient reviews (ratings). As shown in Figure 1, the initial search identified 2837 articles. After reviewing the titles and abstracts to determine relevance and removing duplicates, 90 articles were further reviewed by reading full texts. A total of 48 articles that met the inclusion and exclusion criteria were identified for detailed review. Next, we searched the reference sections of the 48 articles and consulted experts in the field to identify additional articles by hand search, resulting in 26 additional articles for review. After removing duplicates, we identified 69 articles or 63 studies to include in the review. Articles that reported findings based on the same data source, similar design, and research questions were counted as one study. The systematic review protocol was registered in PROSPERO: International Prospective Register of Systematic Reviews.

### Data Extraction and Synthesis

A total of 2 researchers independently reviewed all articles and extracted the following information using a predesigned table: authors and publication year, study time and location, PRWs used in the study, type of providers being studied, number of PORs and providers being analyzed, study design and analytical approach, and key findings. The intercoder reliability, calculated by using Cohen kappa, was 0.86. In the key finding analysis, the researchers listed the bullet points of major findings from each study and discussed the discrepancies until a consensus was reached. Owing to the heterogeneity of the studies, no study appraisal was carried out. This review was not focused on a single health outcome; instead, we aimed to identify and synthesize available POR studies, and no meta-analysis was conducted.

## Results

### Study Time and Location

A total of 63 studies (69 articles) were included in the reviews (Multimedia Appendix 1) [1,3,10,11,25-85]. Although PRWs have been available for more than 2 decades, the earliest study on PORs was published in 2009 [25], and most of the studies (61/63, 96.8%) were published after 2010. Out of 63 studies, 48 were conducted in the United States, including Puerto Rico, 5 in Germany [26-31], 3 in the United Kingdom [32-34], 3 in China [35-38], 3 from the Netherlands [39-41], 1 from Australia [42], and 1 from Canada [86] (Table 1).

**Table 1:** Study locations.

Country	Statistics, n (%)
United States	48 (76.2)
Germany	5 (7.9)
United Kingdom	3 (4.8)
China	3 (4.8)
The Netherlands	3 (4.8)
Australia	1 (1.6)
Canada	1 (1.6)
Total	64^a^

^a^One study was conducted in both China and the United States.

**Figure 2:**
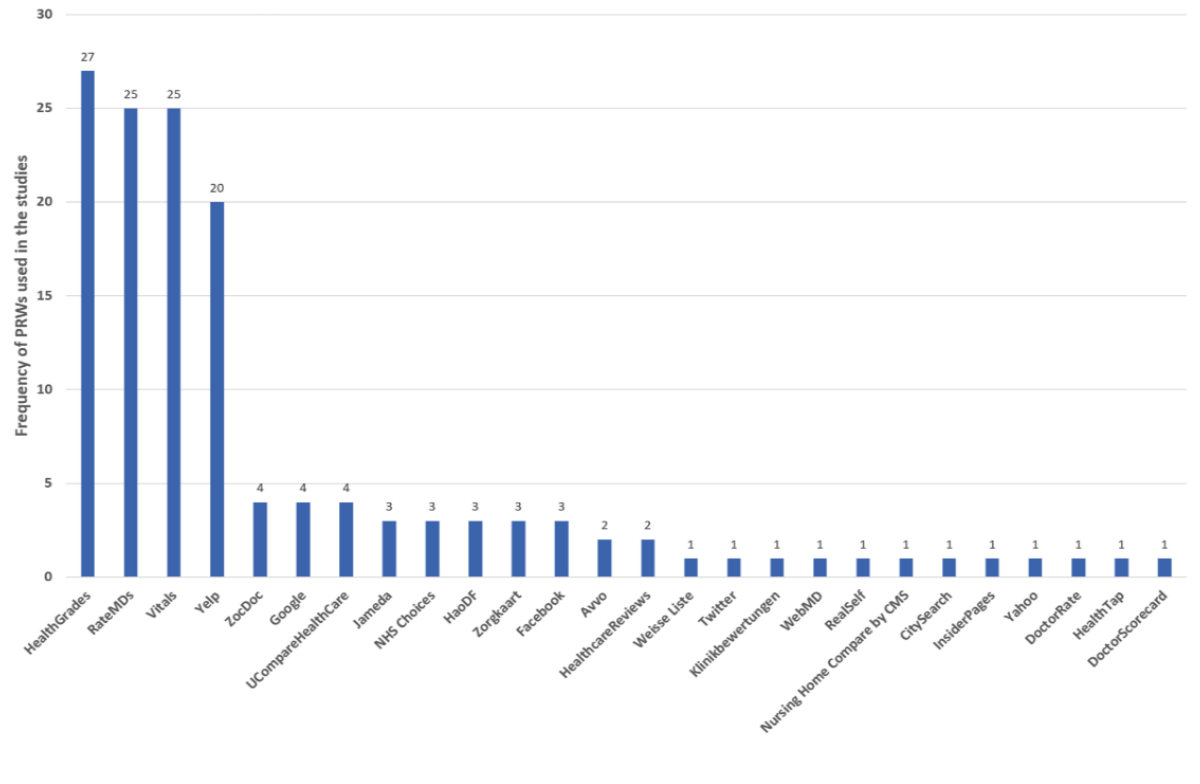
Patient review websites (PRWs) used in the studies.

### Patient Review Websites

Most studies (36/63, 57.1%) retrieved PORs from multiple PRWs with 27 studies (27/63, 42.9%) using a single PRW for data analysis. Some earlier studies googled “patient review” or providers’ names to retrieve PORs, and the most popular or promoted PRWs emerged through such an online search. The PRWs used in these research studies varied across countries. For example, in the United Kingdom, most physicians and hospitals were rated on the National Health System Choices website, which was a single PRW used in the studies from the United Kingdom [32-34]. The most popular PRW in Germany was Jameda [28-31], whereas HaoDF was used in China [35-38]. In the United States, a large number of PORs have accrued on the most popular PRWs including generic consumer review sites such as Yelp, Yahoo, and Google, as well as specialized PRWs such as RateMDs, HealthGrades, and Vitals (see Figure 2).

Studies that compared multiple PRWs found a low correlation between these sites [43,44]. For example, Nwachukwu et al reported that the correlations (*r*) between PRWs were 0.32 approximately 0.51, *P*<.001[43]. Physicians on one PRW were rated differently on other PRWs, whereas no PRW contained all consensus core domains of quality measures [45]. Some studies questioned the reliability of PRWs given that most physicians only have a very small number of ratings [10,46].

**Figure 3:**
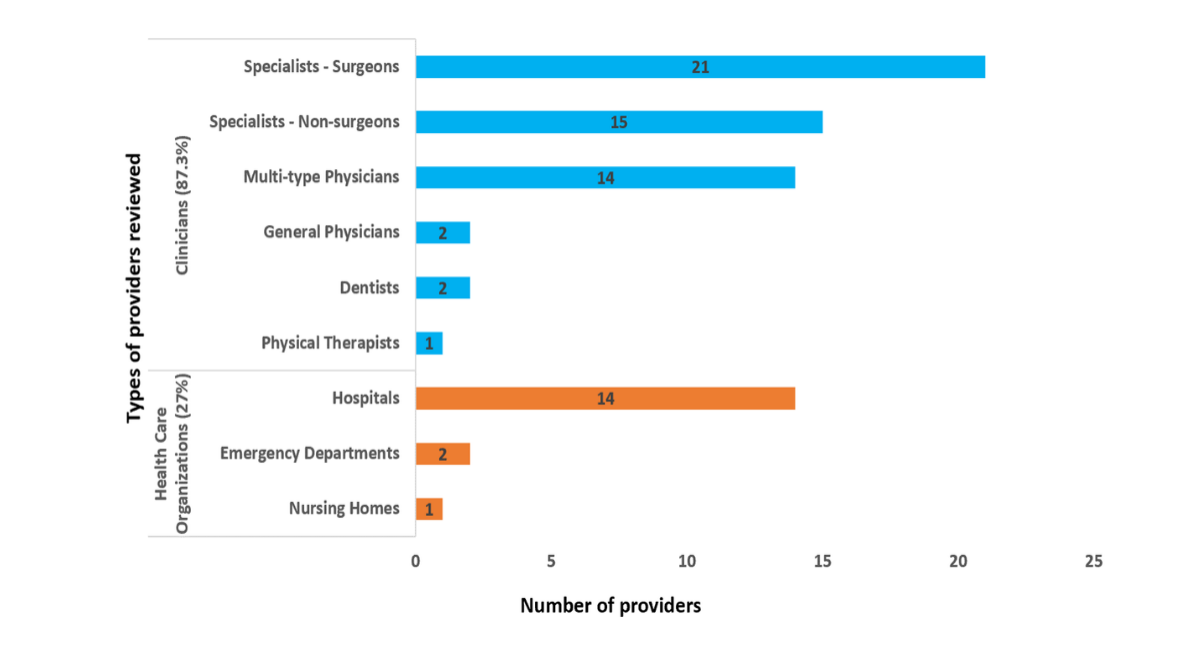
Various types of providers reviewed.

### Types of Providers Reviewed

Out of the 63 studies, 17 (17/63, 27%) reported PORs of HCOs, including hospitals, urgent care centers (emergency departments), and nursing homes [27,32,34,39,40,44,47-53,87,88], and 55 (55/63, 87.3%) were focused on clinicians. Of the 55 studies that reported PORs of clinicians, 14 (14/55, 25.5%) included multi-type physicians (general practitioners and specialists), 2 (2/55, 3.6%) were focused on general practitioners [33,54,55], 1 (1/55, 1.8%) reported physical therapists [88], 2 (2/55, 3.6%) reported dentists [30,88], and the remaining 36 (36/55, 70.6%) were focused on specialists, including surgeons, dermatologists, urologists, Ob/Gyns. Of these 36 studies on specialists, 21 (21/36, 58.3%) were focused on various types of surgeons (see Figure 3).

### Number of Providers Reviewed and Number of Ratings

The number of health care providers reviewed ranged from 20 to 212,933 with a median of 600. The number of PORs analyzed ranged from 30 to 2,685,066 with a median of 5439. The number of PORs included in the analyses has grown substantially in the past 9 years.

Not all physicians had an online rating. In Germany, only 37% of physicians were rated online [29]; a recent study in 3 metros of the United States reported that 34% of physicians did not have any PORs and most physicians still had no more than 1 review [10]. Even among those physicians with online reviews, the number of PORs varied significantly across specialties. For example, 96% of cardiologists in the United States were rated online [56]; 25% of the hospitals included in Hospital Consumer Assessment of Healthcare Providers and Systems (HCAHPS) had Yelp ratings [51]. Specialists were twice as likely to be rated online than general practitioners [26]; radiologists and pathologists were least likely to be rated online [42].

### Study Design and Analytical Approaches

A considerable number of studies (27/63, 42.9%) were descriptive and reported only frequency analyses, including the average numbers of ratings per provider, the percentages of providers that have been reviewed online, and the mean scores of PORs. Studies that focused on HCOs and specialists typically identified the providers from a directory before searching for their PORs. In contrast, studies that focused on all types of providers typically retrieved PORs directly from PRWs without a preselected list of providers.

A total of 19 (30.2%) studies analyzed the narrative comments of PORs. Previous studies in this regard used traditional qualitative methods to retrieve major themes from these comments [3,25,32,54,55]. Recent studies have applied more advanced techniques such as natural language processing (NLP). For example, topic models, such as Latent Dirichlet Allocation, have been used as an efficient tool to automatically cluster POR comments by topics [35,36,38,50,87]. The use of such advanced analytical methods enabled content analysis of hundreds of thousands of narrative comments.

More than half of the studies (n=38/63, 60.3%) employed a comparative design. They typically compared PORs with (1) traditional surveys of patient experience [27,34], (2) providers’ characteristics [28,29,33,34,57,58], (3) clinic outcomes such as patients’ readmission rates or mortality rates [52,56,59], and (4) traditional “golden standards” of health care quality indicators (eg, HCAHPS structural and quality of care measures) [39,44,45,47,50,53]. Furthermore, 1 study compared PORs between China and the United States [38] and 2 studies compared PORs across different PRWs [43,44].

### General Findings of Patient Online Reviews

Most patients made positive comments about their providers and would recommend their providers to families and friends. Out of 63 studies, 27 studies (27/63, 42.9%) reported mean scores of PORs ranging from 2.37 to 4.51 (out of 5) with a median value of 4 and a mean value of 3.89 (unweighted).

The studies that analyzed the patients’ narrative comments found that these comments covered the entire health care encounter of the facility and staff [54,55], including physicians’ demeanor, staff friendliness, empathy, and cost [60,61]; patients also cared about the ease of scheduling, time spent with patients, and wait time [62].

### Relationship of Patient Online Reviews and Providers’ Characteristics

The existing studies that compared PORs with characteristics of providers found that physicians with higher ratings had the following characteristics: (1) female, young age [29,43,46]; (2) more online presence [58]; (3) board-certified with extensive training experiences and graduated from a highly rated medical school [1,89]; (4) active status and years in practice [63]; (5) specialties [37,88]; and (6) locations [86]. However, some studies found no interactions between PORs and either genders, regions, or academic proclivity [46,64,65]. Furthermore, 1 study found surgeons with higher volume of procedures had higher POR ratings and better comments [59]. Patient characteristics also affected PORs. For example, female, seniors, and patients covered by private insurance were more likely to provide positive PORs [27,30].

### Relationship of Patient Online Reviews and Traditional Patient Surveys

As summarized in Table 2, convergent findings suggested a strong association between PORs and traditional patient satisfaction surveys. For example, several studies found moderate-to-high degrees of correlation between PORs and HCAHPS patient experience measures [31,33,34,53,66] and the Press Ganey Medical Practice Survey for patient satisfaction [67], respectively. Content analysis studies also reported a considerable overlap between the narrative comments of PORs and thematic domains of HCAHPS surveys [47,50,51]. Some of these studies also identified additional domains not included in HCAHPS surveys. [50,51]. Similar findings of correlation tests were reported from studies in Germany [31] and the United Kingdom [33,34]. Furthermore, 2 studies from the Netherlands reported that hospitals under supervision or inspection from authorities had lower POR ratings [39,40].

### Relationship of Patient Online Reviews and Clinical Outcomes and Other Quality Measures

Table 2 also includes the summaries of the relationship between PORs and clinical outcomes and other quality measures. Most of the 9 studies on the relationships of PORs and clinical outcomes reported weak or no relationship [11,31,33,52,53,56,66,68,69]. For instance, a study of PORs on cardiologists found no correlation (Spearman ρ=−0.06; *P*=.13) between PORs and mortality rates following the coronary artery bypass surgery [56]. Similarly, Greaves et al found a weak correlation between PORs and clinical outcomes of providers in the United Kingdom (Spearman ρ=−0.18 approximately 0.18; *P*<.001) [33]. By contrast, Bardach et al compared PORs of hospitals in Yelp with quality measures from HCAHPS and found that higher scores of PORs were associated with better clinical outcomes, including lower mortality and readmission rates [53]. Studies also reported significant but low degrees of association between PORs and (1) patient likelihood of visiting their primary care physicians within 14 days of discharge [11], (2) cost of care [11,66], (3) 30-day readmission and length of stay [69], and (5) other hospital level CMS quality measures [44].

**Table 2:** Studies that compare patient online reviews with traditional healthcare quality indicators.

Study	Comparator measures (patient surveys, clinical outcomes, or other quality measures)	Comparison methods and results
Greaves et al, 2012 [33]	(1) Mailed-based patient surveys. (2) Clinical outcomes from the National Health Service (NHS) Information Center and NHS Comparators (eg, The proportion of patients with diabetes receiving flu vaccinations, proportion of hypertensive patients with controlled blood pressure, proportion of diabetic patients with controlled HbA1C, percentage of low-cost statin prescribing, cervical screening rate, admission rates for ambulatory care sensitive conditions, and the proportion of achieved clinical Quality and Outcomes Framework (QOF) points from available points. N (POR)=16,592, N (physicians)=4934.	(1) ρ =0.37~0.48, *P*<.001 for Pearson correlation of POR and survey. (2) ρ=–0.18~0.18, *P*<.001 for the correlation of POR and clinical outcomes.
Greaves et al, 2012 [34]	Traditional survey of patient experience. N (POR)=9,9997, N (physicians)=146.	ρ=0.13~0.49, *P*<.001 for Pearson correlation of POR and survey.
Segal et al, 2012 [59]	Volume of surgeries. N of POR=588, N of surgeons=600.	High volume surgeons have higher mean values of PORs than low-volume surgeons, but effect size was weak.
Bardach et al, 2013 [53]	(1) Overall hospital ratings on HCAHPS. (2) Hospital individual HCAHPS domain scores (eg, nurse communication, pain control). (3) Hospital 30-day mortality and hospital 30-day readmission rates. N (POR)=3796, N (hospitals)=962.	Pearson correlation (n=270), ρ=0.49, *P*<.001 for 3 out of 4 measures. Higher ratings were associated with lower mortality and readmission rates.
Wallace et al, 2014 [11]	(1) Likelihood of patient visiting their primary care physician within 14 days of hospital discharge. (2) Health care expenditure. N (POR)=58,110, N (physicians)=19,636.	(1) Regression model for sentiment generated from POR comments and the comparator r^2^=.21, *P*=.03; (2) Regression model for POR rating combined with topics generated from POR comments r^2^=.25.
Glover et al, 2015 [52]	30-day hospital-wide all-cause unplanned readmission rate (HWR). POR=Facebook comments. POR=Facebook comments, N (hospitals)=136.	Independent sample t test (n=315 vs 364), POR=4.15±0.31 vs 4.05±0.41, *P*<.01 more PORs was associated lower HWR.
Emmert et al, 2015 [31]	(1) Quality measures on cost of medication, type 2 diabetes-related intermediate outcome measure, and patient/doctor ratio from German Integrated Health Care Network (QuE); (2) German patient satisfaction survey from QuE. N (POR)=1179 on Jameda, N=991 on Weisse Liste. N (physicians)=69.	(1) Spearman’s rank correlation (n=991) ρ=0.297~.384, *P*<.05 for cost per prescription; ρ=0.478, *P*<.05 for patient with HbA1c-target values; ρ=−0.316~−0.289, *P*<.05 for patient/doctor ratio on Weisse Liste; (n=1179) ρ=0.298, *P*<.05 for cost per case, ρ=0.298~386, *P*<.05 for patient/doctor ratio on Jameda; (2) Spearman’s rank correlation (n=991), ρ=−0.347~−0.372, *P*<.05 for 3 out of 4 measures on Weisse Liste; (n=1179), ρ=−0.391~0.640, *P*<.05 for all measures on Jameda.
Okike et al, 2016 [56]	Risk-adjusted mortality rate. N of POR NA^a^, N (surgeons)=590.	Pearson’s correlation (n=590), r=−.06, *P*=.13.
Bardach et al, 2016 [51]	Researchers identified HCAHPS domains. N (POR)=244 (narratives), N (hospitals)=193.	Content analysis (139/244, 57% of POR comments mentioned HCAHPS domains).
Kilaru et al, 2016 [47]	HCAHPS inpatient care surveys. N (POR)=1736, N (Emergency Departments)=100.	Content analysis. Considerable overlaps in theme of PORs and HCAHPS domains.
Ranard et al, 2016 [50]	Researchers identified HCAHPS domains. N (POR)=16,862, N (hospitals)=1352.	Content analysis. POR comments covered 7/11 HCAHPS domains and introduced 12 new domains not existing in HCAHPS.
Emmert et al, 2018 [44]	Hospital-level quality measures by the CMS. N (POR)=1000, N (hospitals)=623.	(1) Spearman’s correlation ρ=±0.143, *P*<.05 for 13 of 29 measures; (2) Spearman’s correlation ρ=±0.114, *P*<0.05 for 7 of 29 measures, indicating weak association.
Trehan et al, 2018 [68]	Total knee replacement (TKR) outcomes: infection rate, 30-day readmission rate, 90-day readmission rate, revision surgery. N of POR NA, N (surgeons)=174.	Kruskal–Wallis one-way analysis of variance one-way analysis of variance (one-way ANOVA on ranks) showed no correlation.
Campbell et al, 2018 [66]	1) HCAHPS patient satisfaction measures; 2) HCAHPS hospital-wide 30-day readmission rate; 3) Medicare spending per beneficiary ratio. N of POR NA, N (hospitals)=136.	(1) Multivariable linear regression (n=136), r^2^=.16~.5, *P*<.05 for 21 of 23 measures; Pearson’s correlation (n=136), r=.27~.61, *P*<.005 for 19 of 23 measures; (2) Multivariable linear regression r^2^=−.58, *P*<.10 for readmission rate; (3) Multivariable linear regression r^2^=−.006, *P*<.731 for Medicare spending per beneficiary. Overall weak association.
Jarari et al, 2018 [71]	Nursing Home Compare (NHC) website quality measures.	POR rating was significantly different from NHC rating.
Chen et al, 2018 [67]	Press Ganey Medical Practice Survey for patient satisfaction. N of POR NA, N (physicians)=200.	Pearson’s correlation (n=226), r=.18, *P*<.001.
Daskivich et al, 2018 [69]	Specialty-specific performance scores (adherence to Choosing Wisely measures, 30-day readmissions, length of stay, and adjusted cost of care), primary care physician peer-review scores, and administrator peer-review scores.	Multivariable linear regression (n=30) r=−.04, *P*=.04.

^a^NA: not available.

## Discussion

To the best of our knowledge, this is the first systematic review of studies on PORs. The 63 studies included in this review reflect a decade of peer-reviewed publications on PORs from 6 countries; the study design and key findings have been summarized. Earlier studies tended to report on characteristics of PORs whereas later studies tended to compare PORs with traditional patient surveys or clinical outcomes.

### Principal Findings

Our summaries of the existing 63 studies on PORs revealed that the number of health care providers (including clinicians and HCOs) being reviewed represented only a small number of the total health care workforce. The number of reviews per clinician varied from zero to hundreds, indicating a very skewed distribution in these PORs. As compared with general practitioners, specialists, especially surgeons, were more likely to be reviewed and included in the analyses of PORs. Overall, the online ratings and comments were positive. Only a small number of studies compared the correlations between PORs and patient satisfaction and clinical outcomes. These studies suggested that PORs were highly correlated to the “patient experience” measured by traditional patient surveys. Nevertheless, there were inconclusive findings on whether PORs were inconsistent with traditional measures of clinical outcomes. Notably, reviewed studies have identified several domains of patient experience that were not covered by the traditional patient surveys, for example, HCAHPS [50,51].

The current literature on PORs suggests a relatively new but fast-growing field. The number of published studies was small when compared with the exponential growth of PORs. Therefore, we have made the following recommendations for future studies on PORs.

First, studies with rigorous design, longitudinal nature, and larger samples are needed. POR studies present challenges of data acquisition and processing because of the nature of large and heterogeneous online data. The latest Web crawling techniques have enabled efficient retrieval of large quantities of POR data. Advanced analytical techniques such as machine learning and NLP can be employed to expedite large-scale analysis of PORs.

Second, most existing studies are focused on specialists in metropolitan areas [10,70-72]; more studies are needed to understand other disciplines of health care providers and those who serve in nonmetro areas. Studies that identify consumer-based assessments for underrepresented types of HCOs, such as nursing homes, public health services, and substance treatment centers, are minimal or missing in the literature. There was only 1 study that reported PORs for nursing homes [49]. Many of these HCOs serve vulnerable populations who are not typical PRW users, but their family caregivers and other advocates may also provide valid PORs.

Third, we anticipate more studies that go beyond the simple descriptive analysis and test theory-based hypotheses to provide more clinical and policy implications. In recent years, we have observed emerging studies that compared PORs with traditional measures of patient experience and clinical outcomes. However, the current literature is limited in terms of a lack of consistent POR reporting and insufficient advanced statistical analyses of POR data and their relationship with quality measures. We call for more empirical studies with meaningful hypotheses, rigorous design, and appropriate data analytics.

Finally, we have observed that PORs have begun clustering on a small number of popular PRWs (Figure 2). With the recent announcement of Amazon’s entrance into health care [90], online reviews by health care consumers may become even more clustered. Whether and how the clustering of PORs on the growing dominance of commercial PRWs would affect consumer health behaviors and health care quality remains unstudied.

### Policy Implications

The growing body of literature on PORs indicates its increasing importance in patients’ decision making, which provides policy and practice implications for health care providers, patients, PRW owners, and policy makers.

Notably, health care providers should not underestimate the importance of PORs. Instead, they should recognize the importance of PRWs for their “digital brand” and stay aware of the PORs posted to popular PRWs [91]. Physicians can use anonymous PORs for the evaluation of patient satisfaction and assessment of patients’ need. In addition, friendly and personalized responses to PORs may enhance positive patient-provider communication [19].

From a consumer’s perspective, patients need to know that only a small number of physicians have been reviewed online and the average rating score for a physician might not be sufficient for choosing a doctor as assumed, given the tendency of consumers to provide feedback on experiences that are unusually positive or negative. As posting the health care experience becomes more commonplace, we anticipate a “consumer’s guide” to help patients navigate the PORs and make more informed choices [92,93].

For PRW owners, as PORs are often unstructured, not adjusted for risks, and unverifiable, they should take more social responsibilities by adding design components to enable identity authentication, to remove inflammatory or abusive comments, and to assist patients on how to use PRWs to avoid misinformation [3,4,94]. We also call for a consistent rating scheme to facilitate the evaluation of providers using data from various PRWs.

For policy makers, the question of whether PORs can be used as an indicator of health care quality is still controversial; policy makers and health care providers should acknowledge and embrace its increasing importance for patients [7,95]. The PORs can reflect instant feedback of patients’ medical encounters, the context of their ratings, and what they truly value. Some of the constructs of patient experience identified from analyzing PORs can be used to strengthen or complement the current measures of health care quality and to provide rapid recognition of quality perception gaps along with service corrections or other proactive quality interventions when needed [96]. Although we recognize the growing weight of PORs in consumer health behaviors and the potential of applying PORs in improving health care quality, we call for broader collaborations of key stakeholders, including patients, caregivers, health care providers, PRW owners, policy makers, and health services researchers, to engage in conversations and joint efforts to construct a positive patient-provider feedback loop.

Some potential biases need to be noted while interpreting the results from this review. First, this review was focused on the published studies that analyzed PORs, so the findings related to PORs only reflected those published studies but not the whole picture of PORs. Given the vast and ever-growing number of PORs, only a small fraction was studied and published. Second, only a small number of patients would actually provide ratings of their medical encounters. These motivated patients are more likely to be younger, female, living in metropolitans, and spending more time online [4]; thus, there is a potential bias in the existing PORs. These biases are not methodological flaws in conducting the systematic review but require caution when interpreting study findings.

### Limitations

In addition to the above potential biases, we should also note the limitations of the study. Though we tried our best to thoroughly search the major databases, it is possible that some relevant studies were missed. As we concluded the search in January 2019, a few recently accepted papers were not included. Our search was limited to peer-reviewed literature; we may have missed some gray literature that is equally important for the POR research. Additionally, because our review was limited to the literature published in English, the review did not cover articles published in other languages. Finally, because of the heterogeneity in outcome reporting and study design, we did not carry out an appraisal of study quality. The number of PORs ranged from a few dozens to hundreds of thousands and the ratings were based on different scales, so we did not conduct a meta-analysis.

### Conclusions

To conclude, the current body of the peer-reviewed literature on PORs is still small but growing rapidly. We found that overall PORs tended to be positive, and the narratives of PORs have provided insights into multiple domains of patient experience and health care quality. We call for more research on PORs using rigorous design and large samples along with better use of POR information by patients, physicians, and policy makers. We also advocate for recommendations or guidelines of POR use to help patients make informed choices and foster the application of PORs for improving health care quality.

## Appendix

Multimedia Appendix 1 Summaries of published studies on patient online reviews (63 studies consisting of 69 articles).

## Abbreviations

CMS: Center for Medicare and Medicaid Services
HCO: health care organization
HCAHPS: Hospital Consumer Assessment of Healthcare Providers and Systems
NLP: natural language processing
POR: patient online review
PRW: patient review website
