# Metadata and Table Caption Correction: What Do Patients Say About Doctors Online? A Systematic Review of Studies on Patient Online Reviews

**DOI:** 10.2196/14823

**Published:** 2019-07-18

**Authors:** Y Alicia Hong, Chen Liang, Tiffany A Radcliff, Lisa T Wigfall, Richard L Street

**Affiliations:** 1 Department of Health Administration and Policy George Mason University Fairfax, VA United States; 2 School of Public Health Texas A&M University College Station, TX United States; 3 Arnold School of Public Health University of South Carolina Columbia, SC United States; 4 Department of Health Kinesiology Texas A&M University College Station, TX United States; 5 Department of Communication Texas A&M University College Station, TX United States

The authors of “What Do Patients Say About Doctors Online? A Systematic Review of Studies on Patient Online Reviews” (J Med Internet Res 2019;21(4):e12521) made an error in the caption of Table 2. It previously read “Summaries of published studies on patient online reviews (63 studies consisting of 69 articles)” but has now been changed to “Studies that compare patient online reviews with traditional healthcare quality indicators”.

The lead author, Y Alicia Hong, now has an additional affiliation (Department of Health Administration and Policy, George Mason University, Fairfax, VA, United States) in addition to her previous affiliation (School of Public Health, Texas A&M University, College Station, TX, United States). This has bumped the numbering of all other affiliations by one, although affiliations remain the same for all other authors.

Y Alicia Hong is also the corresponding author and wishes to use her new affiliation’s information for correspondence. The previous contact information was as follows:

Y Alicia Hong, PhD

School of Public Health

Texas A&M University

212 Adriance Lab Road

College Station, TX, 77843-1266

United States

Phone: 1 9794369343

Email: yhong@sph.tamhsc.edu

The new contact information is as follows:

Y Alicia Hong, PhD

Department of Health Administration and Policy

George Mason University 4400 University Drive, MS 1J3

Fairfax, VA, 22030

United States

Phone: 1 703 993 1929

Email: yhong22@gmu.edu

The correction will appear in the online version of the paper on the JMIR website on July 18, 2019, together with the publication of this correction notice. Because this was made after submission to PubMed, PubMed Central, and other full-text repositories, the corrected article also has been resubmitted to those repositories.

