# Supplementary material for: What Do Patients Say About Doctors Online? A Systematic Review of Studies on Patient Online Reviews
Source: J Med Internet Res. 2019 Apr 8;21(4):e12521. doi: 10.2196/12521 (PMC6475821; doi:10.2196/12521)
Supplement: Multimedia Appendix 1 [file jmir_v21i4e12521_app1.pdf]

Appendix table 1. Summaries of published studies on patient online reviews (63 studies consisting of 69 articles).

Authors	Time and Location	Rating websites	Type and number of providers reviewed	Number of online ratings and comments	Research design and analytical methods	Key findings
Black et al., 2009 [25]	2009, the US (Dallas, New York City, Chicago, San Francisco).	1 site: RateMDs.	Providers (n=6,101).	16,703 ratings and 15,952 comments.	Qualitative and quantitative analysis of POR.	Mean rating=3.7~4.0/5.
Lagu et al., 2010 [3]	2009, the US (Boston).	33 sites.	Physicians (n=81) out of 300 random samples.	190 reviews.	Qualitative and quantitative analysis of POR.	Positive reviews: 88%. Generalists & specialists did not differ in number or nature of reviews.
Kadry et al., 2011 [75]	2010, the US.	10 sites from Google Trends; mostly from HealthGrades, Vitals, Yelp.	Multiple specialties (n=23) in 25 metros.	4,999 online ratings.	Calculated online ratings.	Average rating was 3.85/5; 2/3 patient reviews were favorable.

Gao et al., 2012 [1]	2005-2010, the US (Virginia).	1 site: RateMDs.	Physicians with ratings on RateMDs and registered in Virginia Board of Medicine.	386,559 physician ratings.	Ratings of 4 domains: staff, punctuality, helpfulness, and knowledge on scale of 1~5.	Mean online rating=3.9/5. OB/Gyn were twice likely to be rated than other physicians. Higher ratings: recently graduated, board-certified, from highly rated medical schools, and no malpractice claims.
Emmert et al., 2012 [26]	2012, Germany.	8 sites (German) identified through Google and Yahoo.	Physicians (n=53,585).	127,192 ratings; 107,148 patient evaluations.	Categorized POR according to structure/process/outcome model.	Mean number of rating=2.37. Most rated specialties: orthopedists, dermatologists & gynecologist; 2/3 POR were “very good”.
Greaves et al., 2012a [33]	2009-2010, the UK (England).	1 site: NHS Choices.	Family practices (n=4,934).	16,952 ratings.	Compared PORs with patient surveys and clinical outcomes obtained from NHS Information Center and NHS Comparators.	64% of patients would recommend their GP. Correlation of POR & survey = 0.37~0.48; correlation of POR & clinical outcomes=0.18.
Greaves et al., 2012b [34]	2009-2010, the UK (England).	1 site: NHS Choice.	Acute general NHS hospital trusts in England (n=146).	9,997 ratings.	Compared POR with paper-based patient surveys.	67.4% of patients would recommend the hospitals. Correlation of POR & paper-based survey=0.31~0.49.

Segal et al., 2012[62]	The US.	9 sites: Avvo, HealthGrades, RateMDs, Vitals, Google, CitySearch, InsiderPages, Yahoo, Yelp.	Surgeons of bariatric, lumbar, total knee replacement surgery (n=600).	588 ratings.	Compared online ratings with surgical volume as a proxy for clinic outcome and safety.	91.2% of surgeons had POR. High volume surgeons received more ratings than low volume surgeons and higher proportion of praises, but effect size was weak.
Bardach et al., 2013 [56]	2011, the US.	1 site: Yelp.	Hospitals (n=962) with Yelp ratings.	3796 ratings.	Compared POR with data from HCAHPS[3].	25% of HCAHPS hospitals had yelp rating. Correlation of Yelp rating and HCAHPS=0.49. Higher rating was associated with lower mortality & readmission rates.
Lagu et al., 2013 [32]	The UK.	1 site: NHS Choices.	Hospitals (n=20) randomly selected from 264 hospitals.	200 reviews.	Qualitative & quantitative analyses of POR and patients' surveys.	Most comments were positive; 62% of comments were about technical aspects of hospital care; hospitals replied to 56% of patient reviews.
Lopez et al., 2012 [57]; Detz et al., 2013 [58]	2012, the US (Atlanta, Chicago, New York	2 sites: Yelp, RateMDs.	Primary care physicians (n=445) from RateMDs (n=397) and Yelp (n=315).	712 reviews.	Qualitative analysis of POR.	63% of POR were positive. Care encounter reached past the physician: staff, access, & convenience.

	City, San Francisco).					
Ellimoottil et al., 2013 [68]	The US.	10 sites, but mostly from HealthGrades, Vitals, RateMDs, and Avvo.	Urologists (n=500) randomly selected from database. 398 of them had at least 1 rating.	NA	Calculated numerical ratings, categorized qualitative reviews as positive/negative.	80% of urologists had ≥1 rating; mean number of rating = 2.4; 86% of POR have no difference in POR between genders, regions, & city sizes.
Wallace et al., 2014 [11]	The US.	1 site: RateMDs.	US doctors (n=19,636).	58,110 reviews.	Probabilistic generative model (f-LDA) that captured latent sentiment across aspects of care.	Mean rating = 3.7/5. Median word count in review = 41. Generative models show POR correlated with measures of health care quality & expenditure.
Drevs & Hinz, 2014 [27]	2011, Germany.	1 site: klinikbewertungen (German)	Hospitals.	822 from 695 patients.	Analyzed POR by patient characteristics.	Hospitals of self-choice received more positive reviews than those of other-directed.
Emmert et al., 2014[28]; Emmert &	2012, Germany.	1 site: Jameda (German)	Physicians (n=53,585)	127,192 ratings of 53,585 physicians	Compared patients' ratings with physicians' characteristics.	37% of German outpatient physicians were rated. Mean number of POR = 2.37. Specialists were more likely rated than general practitioners

Meier, 2013[29]				from 107,148 patients.		or lab-based physicians. 2/3 ratings positive. Female physicians had better ratings than male.
Atkinson, 2014 [42]	2013, Australia.	3 sites: RateMDs, DoctorRate, Health care Reviews.	Physicians.	4,157 ratings.	Frequency analysis of POR.	Mean number of POR = 3.4/5; mean score of rating = 3.9/5. Only 4.4% of registered doctors were rated; 47.3% of POR were for general practitioners. Radiologists & pathologists were least likely rated.
Sobin & Goyal, 2014 [84]	2013, the US (Northeast)	2 sites: HealthGrades, Vitals.	Otolaryngologists (n=281) in 25 academic programs in Northeast of US.	NA	Compared POR ratings using analysis of variance (ANOVA).	Mean rating = 4.4/5 & 3.4/4. Number of ratings per physician = 4.7. Most POR were positive.
Bakhsh & Mesfin, 2014 [65]	2012, the US (St. Louis, MO).	4 sites: HealthGrades, Vitals, RateMDs, UCompareHealth care.	Orthopedic surgeons (n=131).	2,185 reviews.	Frequency and regression of POR by categories.	Mean rating = 81.8/100. Ratings varied across sites. Higher rating: ease of scheduling, time spent with patient, wait time, surgeon proficiency, & bedside manner.

Emmert et al., 2015a [30]	2013, Germany.	1 site: Jameda (German).	Dentists (n=23,902).	76,456 ratings from 72,758 patients.	Median test and the Kendall tau-b test.	44.5% of German dentists were reviewed; 90% of those reviewed received positive ratings. Better ratings were given by female, older patients, or those covered by private insurance.
Glover et al., 2015 [55]	2014, the US.	1 site: Facebook.	Hospitals (n=315) with readmission rate better than national average; Control hospitals (n=364) below national average.	NA	Retrospective case control study. Compared POR with Hospital Compare Metric, especially 30-day unplanned readmission rates.	Mean ratings = 4.05~4.15/5. Better POR was associated with lower 30-day readmission rate. Aggregate measures of patient satisfaction on social media correlated with traditional measures of hospital quality.
Emmert et al., 2015b [31]	2011-2013, Germany.	2 sites: Jameda, Weisse Liste (German).	Physicians (n=65) from German Integrated Health Care Network (QuE).	1,179 ratings on Jameda; 991 ratings on Weisse Liste.	Compared 21 structural and quality of care measures with POR.	7 out of 21 indicators were strongly correlated between survey & POR. Mean rating=16.80 per practice. Weisse Liste rating = 3.9~4.4/5. Jameda rating = 1.6~1.8/1~6[(2)]. Big differences between PRWs.
Frost & Mesfin, 2015 [74]	The US (30 metros).	7 sites: Vitals, HealthTap, HealthGrades,	Orthopedic surgeons (n=525).	1,562 reviews.	Descriptive statistics on ratings.	Mean rating = 71.4/100. No difference in rating between genders and regions. Surgeons

		Yelp,RateMDs, DoctorScorecard, Health care Reviews.				with practice time of 6-10 years had higher ratings than others.
Lewis et al., 2015 [85]	The US (Southern California).	3 sites: HealthGrades, Vitals, UCompareHealth Care.	Board-certified plastic surgeons (n=263) in the region.	NA	Searched ratings by surgeon's names. Calculated POR.	Mean rating = 85%. Number of POR = 0~222 with mean value of 11 POR per PRW; 97% had ≥ 1 ratings. No relationship of number of POR and mean rating.
van de Belt et al., 2015 [41]	Netherlands.	3 sites: Facebook, Twitter, Zorgkaart (Dutch).	All health care providers.	NA	Searched for incident- or risk-related PORs on social media & PRWs.	Only PORs on PRW added additional values to health care quality check, other social media sites did not.
Hao & Zhang, 2016 [35]; Hao, 2015 [36]	2006-2014, China.	1 site: HaoDF (Chinese).	Chinese doctors (n=75,000) from Internal Medicine, OBGYN, Pediatrics, & Chinese Medicine.	730,000 ratings and reviews.	Extracted hidden topics using LDA.	2/3 of doctors received ≥ 2 reviews. Some doctors received > 500 reviews. Most popular topics were experience of finding doctors and treatment.
Nwachukwu et al., 2016 [44]	2015, the US.	3 sites: HealthGrades,	Sports medicine surgeons (n=275) from American	NA	Compared POR across sites. Multivariate	Mean rating = 4.0/5. Higher ratings were associated with female, fewer years of practice.

		RateMDs, Vitals.	Orthopedic Society for Sports Medicine member directory.		regression of good ratings on covariates.	Low to moderate correlation between rating sites (*r* = .32~.51).
Okike et al., 2016 [59]	2015, the US (CA, MA, NJ, NY, PA).	4 sites: HealthGrades, Vitals, RateMDs, UCompareHealth Care.	Cardiac surgeons (n=590) in the 5 states listed in state reports.	NA	Compared POR with 30-day risk-adjusted mortality rate following coronary artery bypass surgery.	Mean rating = 4.4/5. 96% of cardiac surgeons were rated online. No correlation between rating and age-adjusted mortality rates.
Bardach et al., 2016 [54]	2013, the US.	1 site: Yelp.	HCAHPS hospitals with > 100 PORs on Yelp (n=193)	244 narrative reviews with more than 10 words.	Thematic analysis of narrative reviews and compared with HCAHPS domains.	POR rating was positively associated with HCAHPS rating. 50% of reviews were from patients, 38% from friends or family. Only 57% of reviews mentioned any HCAHPS domain. Additional salient domains were: Financing, unexpected out-of-pocket costs, difficult interactions with billing departments; system-centered care; & perceptions of safety.

Trehan et al., 2016 [61]	2014-2015, the US.	3 sites: HealthGrades, RateMDs, Vitals.	Hand surgeons (n=245) randomly selected from American Society for Surgery of Hand member directory.	NA	Compared POR with physicians' characteristics.	Mean rating = 3.3~4.0/5. 98% of hand surgeons were rated online. Higher rating was associated with higher number of ratings, Castle Connolly Status, & increased online presence.
Riemer et al., 2016 [60]	2015, the US.	5 sites: ZocDoc, Yelp, RateMDs, Vitals HealthGrades.	Dermatologists (n=100).	3,448 ratings.	Mean score of POR and its association with physician characteristics.	Mean rating = 3.5/5. No difference between PORs across PRWs, no difference between genders & specialty training.
Kilaru et al., 2016 [48]	The US.	1 site: Yelp.	Emergency departments from hospitals (n=100) by stratified random sampling.	1,736 reviews.	Compared POR with HCAHPS inpatient care survey. Qualitatively coded review texts.	POR were similar to inpatient surveys, especially PPC[5] & pain control. Major themes specific to ED care were: wait-time & efficiency, decisions to seek ED care, & events following discharge.
Ranard et al., 2016 [53]	2014, the US.	1 site: Yelp.	Hospitals (n=1,352) with Yelp reviews and HCAHPS data.	16,862 reviews.	LDA compared POR with HCAHPS[3] survey data of hospitals.	Median rating=3.2/5. Domains in Yelp reviews covered the majority of HCAHPS domains; Yelp reviews covered additional

						12 domains not covered in HCAHPS.
Kool et al., 2016 [39]	2010-2015, Netherlands.	1 site: Zorgkaart (Dutch).	Hospitals (n=7) under intensified supervision; Other hospitals (n=28) as control.	43,856 online ratings.	Examined the influence of supervision by Health Care Inspectorates on POR of hospitals.	Hospitals under intensified supervision had lower rating (mean=8.2/10) as compared with control (mean=8.5/10).
Smith & Lipoff, 2016 [63]	2015, the US (Houston, Philadelphia, Seattle).	2 sits: Yelp, ZocDoc.	Dermatologists (n=90).	518 reviews on Yelp; 4,921 reviews on ZocDoc.	Qualitative analysis to identify key themes associated with high- and low- scores of POR.	Mean rating = 3.46~4.72/5. Positive POR were associated with physicians who were kind, respectful, thorough, empathetic, & cognizant of cost. Patients reported relying on PRWs to identify dermatologists.
Lagu et al., 2017 [10]	2016, the US 3 metros: (Boston, Portland, Dallas).	28 sites: generated from Google search.	Physicians (n=600) randomly sampled from 3 metros.	8,133 ratings & 1,784 comments.	Mean and median number of reviews per physician per site.	Median number of POR = 7 reviews per physician. 34% of physicians did not have any online review. Commercial PRWs have significant limitations.
Ramkumar et al., 2017 [46]	2016, the US.	5 sites: HealthGrades, Vitals, ZocDoc,	Arthroplasty surgeons (n=556) from top 10	27, 792 ratings; 1/3 of ratings	Compared POR with 7 domains of Patient Experience Domain	Mean rating = 4.3/5. No PRWs contained all Consensus Core domains. No difference in POR

		RateMDs, Yelp.	hospitals with orthopedic surgery.	contained narrative comments.	Items of Consensus Core[1].	between academic & non-academic surgeons.
Kirkpatrick et al., 2017 [47]	2016, the US.	2 sites: HealthGrades, Vitals.	Hand surgeons (n=433).	NA	Number of PORs per physician; mean POR score and its association with physician characteristics.	Mean rating = 8.1/10. Mean POR per surgeon = 13 (0~148). No difference in overall score by region or gender. Older age was associated with lower score. Wait time was not associated with negative POR.
Hao et al., 2017 [38]	2015, the US, China.	2 sites: RateMDs, HaoDF (Chinese).	OBGYN specialists from RateMDs (n=25,016) and HaoDF (n=8,167).	RateMDs: 156,558 reviews. HaoDF: 57,342 reviews.	LDA topic modeling to identify the major topics in positive and negative reviews of those two countries.	PORs reflected difference in health care systems & cultures. Chinese patients focused on medical treatment. American patients focused on recommendation for other patients.
Tran et al., 2017 [50]	2016, the US.	1 site: Yelp.	Health care service providers identified by Yelp (n=2,085).	2,685,066 reviews submitted for 85,901 businesses.	Inferential statistics for the association between PORs and temporal accessibility of health care services	Lower ratings were associated with longer hours during normal working hours on Monday, Saturday, and Sunday, and outside normal working hours on Friday. Higher ratings were

					measured by opening hours.	associated with longer normal working hours on Sunday.
Murphy et al., 2017 [86]	1989-2015, the US (California).	3 sites: Vitals, HealthGrades, RateMDs.	Physicians who were placed on probations (n=410) vs. controls (n=818).	NA	Inferential statistics compared POR ratings by whether a doctor was on probation.	POR ratings were lower for doctors on probation. Lower PORs were associated with probation related to infractions for medical documentation, incompetence, prescription negligence, and fraud.
Emmert et al., 2018 [45]	2015, the US.	1 site: RateMDs.	Hospitals (n=623)	A stratified random sample of PORs (n=1,000)	Correlations between PORs and hospital-level quality measures published by the CMS; content analysis.	Some PORs were associated with CMS quality measures, but overall associations were weak.
Zhang W. et al., 2018 [37]	2016, China (Beijing).	1 site: HaoDF (Chinese).	Physicians from 5 top ranked hospitals in Beijing (n=1,029).	3,012 negative comments only.	Content analysis.	Ob/Gyn and Internal Medicine received the most negative comments. Complaints were related to insufficient consultation time, physician impatience, and perceived poor therapeutic effect. Those accompanying older patients or children, traveling patients were

						less likely to have tolerance for poor medical service.
Agarwal et al., 2018 [49]	2018, the US.	1 site: Yelp.	Emergency departments (ED, n=1,566) and urgent care centers (UCC, n=5,601).	Total=100,949 reviews, ED=16,447 UC=84,502	LDA, Topic models; language analysis.	There were more 5-star UCC reviews than 5-star ED reviews. Themes of POR reviews varied between ED and UC, and among types of providers.
Cloney et al., 2018 [66]	2018, the US.	3 sites: RateMDs, HealthGrades, Vitals.	Neurosurgeons (n=3,054) above 25th percentile of number of reviews.	NA	Compared PORs with physician characteristics and across regions.	Median ratings = 4.11/5, but varied between PRWs, regions, and settings. Higher POR scores were associated with ranking of medical school, recent graduation, and fellowship training completion.
Geletta, 2018 [52]	2016, the US.	1 site: Yelp.	Uniquely identifiable health service providers, including dentists, general practice physicians, specialists, physical	1,569,264 reviews.	Compared PORs across different types of providers	Overall rating = 3.8/5. Rating = 3.16/5 for specialty physicians. Rating = 4.52/5 for physical therapists.

			therapists, and hospitals (n=866).			
Liu et al., 2018 [43]	2005-2013, Canada.	1 site: RateMDs.	Physicians identified by RateMDs (n=57,412)	640,603 ratings.	Descriptive and inferential statistics.	Mean POR ratings = 3.9/5. Mean number of POR = 11.2. Physicians of OB/GYN, family medicine, surgery, & dermatology were more commonly rated. Others in pathology, radiology, genetics, and anesthesia were less represented.
Trehan et al., 2018 [72]	2010-2012, the US (New York State).	2 sites: Vitals, HealthGrades.	High-volume total knee replacement (TKR) surgeons from the Statewide Planning and Research Cooperative System (SPARCS) database from the NYS Department of Health. (n=174)	NA	Inferential statistics for differences in TKR outcomes (eg, infection rates, readmission rates, revision surgery rate, etc) by POR ratings.	PORs were not associated with TKR outcomes.

Campbell & Li, 2018 [70]	2015-2016, the US (New York State)	1 site: Facebook.	Acute care hospitals (n=136)	Facebook page comments. Numbers N.A.	Compared PORs with and (1) HCAHPS patient satisfaction measures, (2) the 30-day all-cause readmission rate, and (3) the Medicare spending per beneficiary (MSPB) ratio.	PORs were positively associated with HCAHPS patient satisfaction measures. No correlation between POR rating and (1) 30-day readmission rate; (2) Medicare spending per beneficiary ratio.
Chen et al., 2018 [71]	2015, the US.	2 sites: Vitals, HealthGrades.	Physicians at a university hospital (n=200)	NA	Correlation between POR rating and patient satisfaction survey	PORs were correlated with the Press Ganey Medical Practice Survey (PGMPS).
Daskivich et al., 2018a [73]	2018, the US.	5 sites: HealthGrades, Vitals, Yelp, RateMDs, UCompareHealth Care.	Specialists (n=78)	30 reviews.	Multivariable linear regression for the relationship between PORs & specialty-specific quality of care performance scores	No significant association between PORs and all quality measures: Choosing Wisely measures, 30-day readmissions, length of stay, adjusted cost of care, physician peer-review scores, administrator peer-review scores
Dorfman et al., 2018 [76]	2011-2016, the US (6 large	3 sites: Google, Yelp, RealSelf.	Top-rated plastic surgeons (n=30).	1,077 PORs on breast	Descriptive statistics; content analysis.	PORs on breast augmentation grew by 42.6% annually on average 2011-2016. Ratings

	metropolitan areas).			augmentation surgeries		were distributed bimodally, with peaks at 5 stars and 1 star. 87.5% PORs were positive. 70% PORs were about aesthetic outcomes compared with 8% about cost.
Johari et al., 2018 [51]	2016, the US (California).	2 sites: Nursing Home Compare (NHC) on CMS website, Yelp.	Nursing homes (n=675).	NA	Compared PORs with nursing home NHC quality measures	POR rating was significantly different from NHC rating.
Donnally et al., 2018a [87]; Donnally et al., 2018b [88]; Donnally et al., 2018c [89]	2017-2018, the US (Florida, Texas).	3 sites: HealthGrades, Vitals, Google.	Registered North American Spine Society (NASS) physicians (n_1 =299; n_2 =210; n_3 =215)	229 PORs; 4,701 patient comments; 215 PORs, respectively.	Compared PORs with (1) physicians' social presence (websites or social media accounts); (2) Physician characteristics: competence, character, likeability, personality vs medial staff, billing, scheduling, wait time, office environment).	(1) Social media presence correlated with number of ratings and comments but did not impact overall scores. PORs across 3 PRW were highly correlated. (2) PORs were negatively associated with older age, longer wait time, and absence of websites. PORs reflected physician likability, staff interactions, billing, and clinic environment.

Randhawa et al., 2018 [90]	2016, the US and Puerto Rico.	1 site: Vitals.	Radiation oncologists accepting Medicare (n=4,443).	NA	Associations of POR overall rating with specific ratings including accurate diagnosis, bedside manner, appropriate time spent with patients, etc.	Mean rating = 4.34/5. Positive PORs were correlated with accuracy of diagnosis, bedside manner, less wait time, and time spent with patients.
Haglin et al., 2018 [67]; Kalagara et al., 2018 [91]	2016-2017, the US.	3 sites: HealthGrades, Vitals, RateMDs.	Registered North American Spine Society (NASS) orthopedic spine surgeons (n=282; 250) selected from a total of 2,817 surgeons.	NA	Associations of overall POR rating with coded themes of patient comments including trustworthiness, experience match, professional competence, etc.	Trustworthiness was the strongest predictor of POR overall rating. Academic proclivity, region of practice, and physician sex has no relationship with trustworthiness.
Zhang J. et al., 2018 [92]	2016, the US.	5 sites: HealthGrades, Vitals, RateMDs, WebMD, Yelp.	Spine surgeons (n=209).	615 ratings.	Compared PORs with physician characteristics	Mean rating = 80/100. Average number of POR per surgeon = 2.96.99.5% of spine surgeon had ≥ 1 rating. Surgeons in academic practice had higher ratings. Surgeons with ≥21 years of practice were rated lower.

Prabhu et al., 2018 [93]	2016, the US.	1 site: HealthGrades.	Radiation oncologists participating Medicare (n=2,679).	NA	Descriptive statistics compared PORs with physician characteristics.	Mean Likely To Recommend (LTR) score rating = 4.51/5. LTR was associated with time spent with patient and level of trust.
Jack et al., 2018 [94]	2017, the US (9 cities)	4 sites: HealthGrades, Vitals, RateMDs, Yelp.	Orthopedic surgeons selected from American Board of Orthopedic Surgery (ABOS) database (n=351)	NA	Descriptive analyses for the number of PORs. Inferential statistics compared the number of PORs with years of practice and regions of practice	Mean number of PORs = 9/10. The number of PORs did not differ by age. Surgeons with less than 10 years of acquiring board certification received more PORs.
Skrzypecki et al., 2018 [69]	2016, the US.	2 sites: HealthGrades, ZocDoc.	Ophthalmologists (n=105).	NA	Inferential statistics compared PORs with doctors' academic performance measured by number of publications and citations or Hirsh index.	Mean POR rating = 4.2/5. The POR rating did not correlate with the number of citations or Hirsh index.
Daskivich et al., 2018b [95]	2017, the US.	1 site: HealthGrades.	Health care providers (n=212,933) identified by	212,933 ratings.	Descriptive and inferential statistics to examine whether distributions of POR	POR ratings were highly left skewed, fell within narrow ranges, and differed by specialties.

			HealthGrades, representing 29 medical specialties, 15 surgical specialties, and 6 allied health professions.		ratings differed across specialties.	
McGrath et al., 2018 [96]	2018, the US (Atlanta, Boston, Chicago, Dallas, Washington DC, Los Angeles, Miami, New York, Philadelphia, and San Francisco).	3 sites: HealthGrades, RateMDs, Vitals.	Physicians with more than 2 reviews, including some from the "America's Top Doctors" list (n=24,579)	223,715 ratings.	Descriptive statistics	POR ratings of four specialties (family medicine, allergists, internal medicine, and pediatrics) were higher among the physicians listed as a peer-reviewed "Top Doctor"

Hendrikx et al., 2018 [40]	2008-2017, the Netherlands (9 regions).	1 site: Zorgkaart (Dutch).	Health care providers (n=4,100).	70,889 ratings.	Inferential statistics compared POR ratings and providers' characteristics including regions.	PORs varied slightly by regions PORs can be used to identify under-performing providers within their regions but not sufficient for policy recommendations.
Goshtasbi et al., 2019 [64]	2018, the US.	5 sites: HealthGrades, Vitals, RateMDs, Yelp, Google.	American Neurotology Society members (n=560).	NA	Content analysis.	POR ratings and comments were highly dependent on patient perceptions of physician competence, caring bedside manner, and office management.
